# Characteristics and risk of chronic graft-versus-host disease of liver in allogeneic hematopoietic stem cell transplant recipients

**DOI:** 10.1371/journal.pone.0185210

**Published:** 2017-09-21

**Authors:** Chien-Ting Chen, Chun-Yu Liu, Yuan-Bin Yu, Chia-Jen Liu, Liang-Tsai Hsiao, Jyh-Pyng Gau, Tzeon-Jye Chiou, Jing-Hwang Liu, Yao-Chung Liu

**Affiliations:** 1 Division of Hematology, Department of Medicine, Taipei Veterans General Hospital, Taipei, Taiwan; 2 School of Medicine, National Yang-Ming University, Taipei, Taiwan; 3 Division of Medical Oncology, Department of Oncology, Taipei Veterans General Hospital, Taipei, Taiwan; 4 Division of Transfusion Medicine, Department of Medicine, Taipei Veterans General Hospital, Taipei, Taiwan; University of Kentucky, UNITED STATES

## Abstract

Chronic graft-versus-host-disease (cGvHD) is a serious complication of allogeneic hematopoietic stem cell transplantation (allo-HSCT). Among various organ-specific cGvHD, the cGvHD of liver is less well-characterized. In this study, we applied the National Institutes of Health 2014 scoring criteria of cGvHD to analyze a retrospective cohort of 362 allo-HSCT recipients focusing on cGvHD of liver. The overall incidence of liver cGvHD with a score of 3 by 1.5 years post-transplant was 5.8% (21/362). Poor outcome, in terms of overall survival (OS), were observed in patients with scores of 3 liver cGvHD, comparing to those with scores less than 3 (hazard ratio [HR] 2.037, 95% confidence interval [CI] 1.123–3.696, P = 0.019). In multivariate analysis, male gender (HR 4.004, P = 0.042) and chronic hepatitis C virus (HCV) infection status (HR 19.087, P < 0.001) were statistically significant risk factors for scores of 3 liver cGvHD. Our results indicate that liver cGvHD with scores of 3 has a grave prognosis following allo-HSCT, and that HCV carrier status and male are risk factors. Early recognition of this devastating complication might help in prompt immunosuppressive therapy and reducing late poor outcome.

## Introduction

Chronic graft-versus-host disease (cGvHD) is a serious complication of allogeneic hematopoietic stem cell transplantation (allo-HSCT) and its incidence rate ranges from 30% to 70% [1]. The consequences of cGvHD include impaired patient quality of life, a greater symptom burden and medical costs, and an extended use of immunosuppressive therapy, and late morbidity and mortality [2–5]. To better analyze the association between the severity of cGvHD and survival outcomes, the National Institutes of Health (NIH) Consensus Conference Working Group first proposed criteriafor the diagnosis and scoring of cGvHD in 2005, which were refined in 2014 [6, 7]. In contrast to traditional classifications that divide cGvHD into limited or extensive types [8], the NIH criteria scores eight major organ systems on a scale of 0–3, which are attributed to global severity assessment scales (mild, moderate, or severe) [7]. The revision included changes in the cGvHD scoring of the skin, lungs, and liver. For liver cGvHD, the new NIH criteria increases the weight of bilirubin levels for categorization, with serum total bilirubin levels of 3 mg/dL and above corresponding to a score of 3. The revision also discarded the day-100 post-transplant cut-off for differentiation of acute and chronic GvHD [7]. Previous studies have validated the implications of NIH scores [1, 8], including for cGVHD of major organs, such as lung [9–11] and skin cGVHD [12–15]. However, for cGvHD of liver there is one prospective study that has used the 2005 NIH criteria to describe liver cGvHD [16]. The report demonstrated worse overall survival (OS) and higher non-relapse mortality (NRM) in patients with jaundice-type cGvHD [16]. In this study, we aimed to use the NIH 2014 scoring criteria to characterize cGvHD of liver from a retrospective cohort data and examine the risk factors for liver cGvHD and the impacts on survival outcome.

## Materials and methods

### Patient’ population

We identified consecutive patients who had undergone allogeneic HSCT between January 2003 and December 2013 at the Blood and Marrow Transplant Center in Taipei Veterans General Hospital in Taiwan. All patients were monitored by December 31, 2014. Patients who survived less than 100 days (N = 83) post-HSCT were excluded. A total of 362 patients were enrolled into analysis, including 42 patients below age 18. This study obeyed the principles of the 1975 Declaration of Helsinki, and was approved by the Institutional Review Board of Taipei Veterans General Hospital in Taiwan (VGH IRB no.: 201703002BC). Informed written consent was waived by the approving IRB. In addition, patient record/information was anonymized and de-identified before analysis.

### Clinical assessments and definitions

To diagnose liver cGvHD, patients were required to have liver dysfunctions with concomitant diagnostic or distinctive features of cGvHD of other organs [7]. Liver dysfunctions were defined as rising serum alanine transaminase (ALT) to more than 3 times or total bilirubin above the upper limit beyond day 70 after transplant [17]. Patients with serum total bilirubin levels above 3 mg/dL were given a score of 3, and the other patients were categorized as non-score 3 in this study. Abnormal liver function test findings caused by severe sepsis or septic shock [18], hemolysis, viral hepatitis B or C, acute liver GvHD, or biopsy proven liver hemochromatosis were identified in the non-score 3 group. If patients had recurrent events of liver cGvHD, the highest score was adopted. Hepatitis B virus (HBV) carrier status was confirmed by the positivity of serum hepatitis B surface antigen, and hepatitis C virus (HCV) carrier by positivity of enzyme immunoassay for anti-HCV [19].

### Transplantation and post-HSCT care

We used low- to intermediate-resolution HLA (human leukocyte antigen) tests to detect six to eight alleles (HLA-A, -B,–DR, and/or -C). Patients were classified as either fully matched or mismatched. Donors were divided into matched sibling donor (MSD), matched/mismatched unrelated donor (MUD), umbilical cord blood (UCB), and haplo-identical donor. MUD, UCB, and haplo-identical donors are categorized as non-MSD in analysis. Conditioning regimens were categorized as total body irradiation- (TBI-, 12 Gy divided into six fractions), Busulfan-, Cyclophosphamide (total 120 mg/kg) -based regimens, and as myelo-ablative or reduced-intensity regimens.

To prevent acute GvHD, we administered 3 mg/kg/day cyclosporine in two split doses, with adjusted trough plasma levels of 100–250 ug/L. In general, cyclosporine was tapered starting 2 months post-HSCT over a 3-month period and may be individualized at discretions of attending physicians. Anti-thymocyte globulin (ATG) were given at a dose of 8mg/kg in 4 days for selected cases transplanted with unrelated or haplo-identical donors. Short-term methotrexate was administered on the first (15 mg/m^2^), third, six, and eleventh (10 mg/m^2^, respectively) days after HSCT. One patient received alemtuzumab for in-vivo T cell depletion. No patients had ever undergone post-transplanted cyclophosphamide or pre-transplant graft T cell depletion. Real-time quantitative polymerase chain reaction was performed weekly to test for cytomegalovirus (CMV). Ganciclovir was administered pre-emptively to patients positive for CMV viremia

The principle protocols, such as GvHD prevention and post-transplant care for allogeneic hematopoietic stem cell transplant recipients in Taipei Veterans General Hospital have been well-defined [20]. Treatment of cGvHD was in general consistent with the guidelines [21, 22]. Systemic steroids, usually methylprednisolone at doses of 1–2 mg/kg/day, were the mainstay treatment. Cyclosporine or other immunosuppressive agents such as mycophenolate mofetil were used on an individual basis when persisted/worsened cGvHD despite steroid treatment.

### Evaluation of transplant risk

Patients with European Group for Blood and Marrow Transplantation (EBMT) risk scores [23] of 3 were considered to be at intermediate risk, those with scores between 0 and 2 were considered to be at low risk, and those with scores between 4 and 7 were at high risk. OS was defined as the duration between transplantation to death or the last follow-up.

### Statistical methods and study endpoints

We retrospectively collected clinical data, including patient age at transplant, diagnosis, recipient-donor gender combination, disease status at transplant, hepatitis virus carrier status, conditioning regimen, incidence of GvHD, transplant type, degree of HLA matching, date of death, relapse, and last follow-up. Only data from the last allogeneic HSCT were obtained.

We first analyzed risk factors associated with liver cGvHD scores of 3. The potential variables included age at HSCT, gender, underlying disease, donor types, transplant types, EBMT risk scores, stem cell sources, donor-recipient gender combination, conditioning regimen, TBI dosage, GvHD prophylaxis regimen, and hepatitis B and C carrier status. In Cox regression univariable analysis, significant factors (P < 0.1) were included in the multivariable model. A P value less than 0.05 was considered statistically significant. In the analysis, we used 30 years of age as the cut-off because it was the median age of the subjects in our study.

We next analyzed survival outcome in patients with score 3 liver cGvHD compared to patients with non-score 3 disease. Eight factors were considered, including age, gender, malignant disease, transplant type, conditioning regimen intensity, transplant number, EBMT score, and liver cGvHD score of 3. Univariate and multivariate Cox regression analysis were used to determine the survival outcome for score 3 liver cGvHD. Because most patients undergoing allo-HSCT had scores of 0 to 1 based on the Eastern Cooperative Oncology Group (ECOG) scale, performance status was not included in our survival evaluation. All analyses were performed using SPSS Statistics for Windows, version 20.0 (IBM Corp., Armonk, NY, USA).

## Results

### Patient characteristics and incidence of score 3 liver cGvHD

A total of 362 patients (median age, 30 years; range, 0.67–67) were categorized into groups with hematological malignancies (N = 308) and non-malignant disease (N = 54). Fifty-nine percent of patients were male. There were 176 MSD (48.6%), 178 MUD (49.2%), and (2.2%) haplo-identical or cord blood donors. The median follow-up time after HSCT was 1,039 days (range: 102–4440 days). A total of 37 patients underwent liver biopsy for differential diagnosis of liver cGvHD, including 33 in the sub-cohort having cGvHD (N = 190), and 4 in the sub-cohort not having cGvHD (N = 172). Among the patients with HCV infection, two patients underwent liver biopsy (Table in S1 Table). The median day of score 3 liver cGvHD occurrence was 147 days (range: 90–546 days) after transplant. The clinical characteristics are shown in Table 1. Among 58 patients with liver cGvHD, 21 cases had a score of 3 (Table 2), and 11 patients progressed from previous scores of 1 or 2.

**Table 1:** Clinical characteristics of study population.

Patient characteristics	Cohort, N = 362
**Median patient age at transplantation, y (range)**	30 (0.67~67)
**Patient Gender, no.(%)**	
Male	213(59)
Female	149(41)
**Diagnosis, no.(%)**	
AML/MDS	145(40)
ALL	75(21)
CML	16(5)
MPN	3(0.8)
CLL	4(1)
Lymphoma	51(14)
MM	13(4)
SAA	49(13)
Other	7(2)
**Transplant type, no. (%)**	
MSD	176(49)
Non-MSD	186(51)
MUD	178(49)
UCB	3(0.8)
Haplo-identical	5(1.4)
**Disease risk, no. (%)**	
Low (EBMT score ≦2)	170(47)
Intermediate (EBMT score 3)	92(25)
High (EBMT score ≧ 4)	100(28)
**Stem cell source, no.(%)**	
Mobilized blood cells	359(99)
**Donor-Recipient gender combination, no. (%)**	
Female to male	78(22)
Others	284(78)
**Conditioning Regimen, no. (%)**	
Busulfan-based	164(45)
TBI-based (12Gy)	118(33)
Cyclophosphamide-based (total 120mg/kg)	49(13.5)
Others	31(8.5)
**Intensity of conditioning regimen, no. (%)**	
Myeloablative	244(67.4)
Reduced-intensity	118(32.6)
**TBI dose in conditioning regimen, no. (%)**	
≦450 cGy	44(12)
≧1200 cGy	121(33)
**GvHD prophylaxis regimen, no. (%)**	
CsA plus MTX	360 (99)
ATG	65 (18)
**Patient with chronic GvHD, no. (%)**	190(52)
**Sites**^+^ **involved with chronic GvHD, no. (%)**	
Skin	43(12)
Lung	25(7)
Liver	58(16)
score 1	5(1)
score 2	32(9)
score 3	21(6)
Eye	72(20)
Mouth	81(22)
GI tract	22(6)
Sclerodermatous feature	15(4)
LONIPCs	21(6)

MDS, myelodysplastic syndrome; MPN, myeloproliferative neoplasm; MM, multiple myeloma; SAA, severe aplastic anemia; MSD, matched sibling donor; MUD, matched/mismatched unrelated donor; UCB, umbilical cord blood; EBMT, European Group for Blood and Marrow Transplant; CsA, cyclosporine A; MTX, methotraxate; ATG, anti-thymocyte globulin; GI, gastro-intestine; LONIPCs, late onset non-infectious pulmonary complications

^+^61 patients had multiple sites (≧3) involvement, another 93 patients with 2 organs involved.

**Table 2:** Characteristics of patients with score 3 liver cGvHD.

Patient	Age (years)	Diagnosis	Donor type	EBMT score	Donor-recepient sex combination	HCV carrier	Time to score 3 liver cGvHD (days)	Relapse (days after HSCT)	Survival outcome (days after HSCT)
1	33	CML	MSD	1	M–M	no	126	no	4352, alive
2	24	ALL	MUD	5	F–M	no	545	yes, 535	580, died of relapse
3	18	SAA	MUD	1	M–M	no	133	no	725, alive
4	46	MM	MSD	3	M–M	no	147	yes, 528	663, died of relapse
5	15	MDS	MUD	2	M–M	no	121	no	156, died of CMV pneumonitis
6	48	CML	MUD	4	F–M	no	102	no	785, died of HBV reactivation
7	41	MM	MSD	6	F–M	no	156	yes, 365	944, died of relapse
8	43	MM	MSD	5	F–F	no	165	no	180, died of PJP infection & intracranial hemorrhge
9	28	CML	MUD	3	F–M	no	147	yes, 902	1694, died of GvHD-related cardiac tamponade
10	16	ALL	MUD	1	M–M	no	121	no	4440, alive
11	27	AML	MSD	1	F–M	no	174	no	1341, alive
12	11	lymphoma	MSD	3	F–M	no	126	yes, 260	369, died of relapse
13	50	ALL	MUD	3	M–F	yes	283	no	352, died of liver cGvHD and sepsis
14	58	AML	MSD	4	F–M	no	289	no	379, alive
15	29	AML	MSD	2	M–M	no	124	no	182, died of lung infection
16	67	AML	MSD	2	M–M	no	90	no	266, died of lung infection
17	56	ALL	MSD	2	M–M	no	339	no	415, died of pneumonia and CMV pneumonitis
18	29	ALL	MSD	3	M–F	no	123	no	3326, alive
19	24	HD	MSD	2	M–M	no	236	yes, 218	582, alive
20	28	CML	MSD	2	F–M	yes	100	no	3811, alive
21	27	AML	MUD	5	F–M	no	236	no	248, died of PJP infection

HCV, hepatitis C virus; HBV, hepatitis B virus; HD, Hodgkin disease; MUD, matched/mismathced unrelated donor; PJP, pneumocystis jiroveci pneumonia

**Fig 1:**
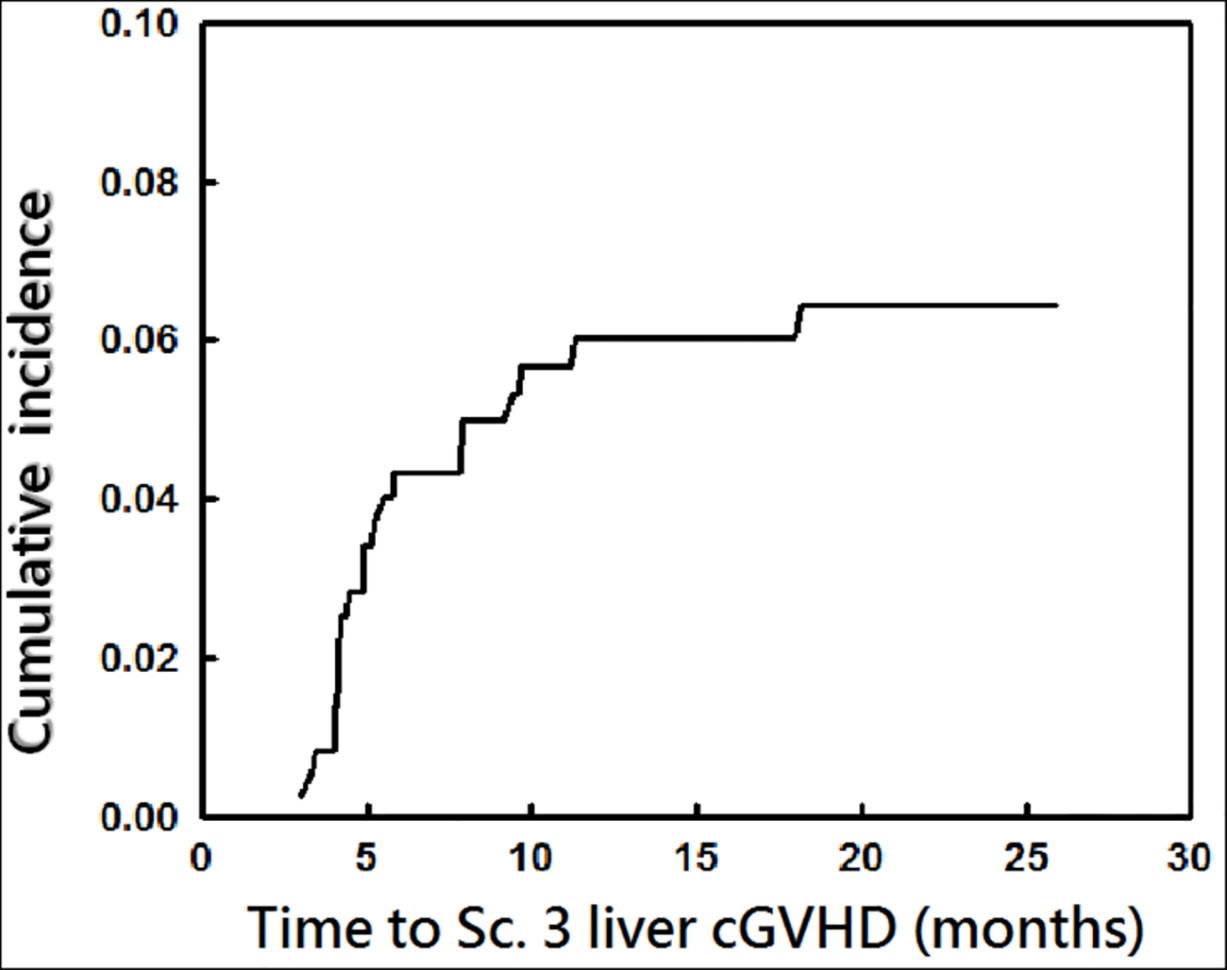
Cumulative incidence of score 3 liver cGvHD after HSCT. The overall cumulative incidence of score 3 liver cGvHD plateaued at 5.8% by 1.5 years.among all patients.

The overall cumulative incidence of score 3 liver cGvHD plateaued at 5.8% (21/362) by 1.5 years among all patients (Fig 1), and 11% (21/190) among patients with cGvHD.

### Risk factors for development of score 3 liver cGvHD

Univariate analysis revealed that younger age (less than 30 years), male gender, female-to-male (F-M) donor-recipient gender combination, and HCV carrier status were significantly associated with liver cGvHD scores of 3. In multivariate Cox regression analysis, male gender and chronic HCV infection remained statistically significant predictive factors, while younger age at transplantation had a trend toward more frequent liver cGvHD scores of 3 (Table 3).

**Table 3:** Risk factors for score 3 liver cGvHD.

Risk factor	Patient (*N*)	Score 3 liver cGvHD		Univariate		Multivariate	
		*N*	%	HR (95% CI)	P	HR (95% CI)	P
**Gender**							
female	149	3	2	1.00 (reference)			
Male	213	18	8.5	4.464 (1.315–15.158)	0.016	4.004 (1.049–15.274)	0.042
**Age**							
≧ 30	237	9	3.8	1.00 (reference)			
< 30	125	12	9.6	2.476 (1.043–5.586)	0.040	2.445 (0.979–6.107)	0.056
**Diagnosis**							
Non-malignant	54	2	3.7	1.00 (reference)			
Malignant	308	19	6.2	1.788 (0.416–7.680)	0.434		
**Transplant type**							
Non-MSD	185	8	4.3	1.00 (reference)			
MSD	176	13	7.4	1.811 (0.751–4.370)	0.186		
**EBMT score**							
≦2	170	10	5.9	1.00 (reference)			
>2	192	11	5.7	1.054 (0.448–2.484)	0.903		
**Conditioning regimen**							
Others	198	9	4.5	1.00 (reference)			
Busulfan-based	164	12	7.3	1.698 (0.715–4.030)	0.230		
**Conditioning regimen**							
Others	238	14	5.9	1.00 (reference)			
TBI-based (**≧**12Gy)	121	6	5.0	0.848 (0.326–2.207)	0.735		
**Conditioning regimen**							
Reduced intensity	118	7	5.9	1.00 (reference)			
Myeloablative	244	14	5.7	0.928 (0.374–2.299)	0.871		
**GvHD prophylaxis**							
Without ATG	288	17	5.9	1.00 (reference)			
With ATG	65	2	3	0.481(0.111–2.080)	0.327		
**Gender combination**							
Others	284	12	4.2	1.00 (reference)			
Female to male	78	9	11.5	2.873 (1.210–6.821)	0.017	1.739 (0.680–4.445)	0.248
**HBV status**							
Non-carrier	313	20	6.4	1.00 (reference)			
carrier	49	1	2.0	0.329 (0.044–2.449)	0.277		
**HCV status**							
Non-carrier	356	19	5.3	1.00 (reference)			
carrier	6	2	33.3	6.684 (1.556–28.705)	0.011	19.087 (3.931–92.672)	<0.001

95% CI, 95% confidence interval; HR, hazard ratio; HBV, hepatitis B virus

### Survival outcome in patients with score 3 liver cGvHD

**Fig 2:**
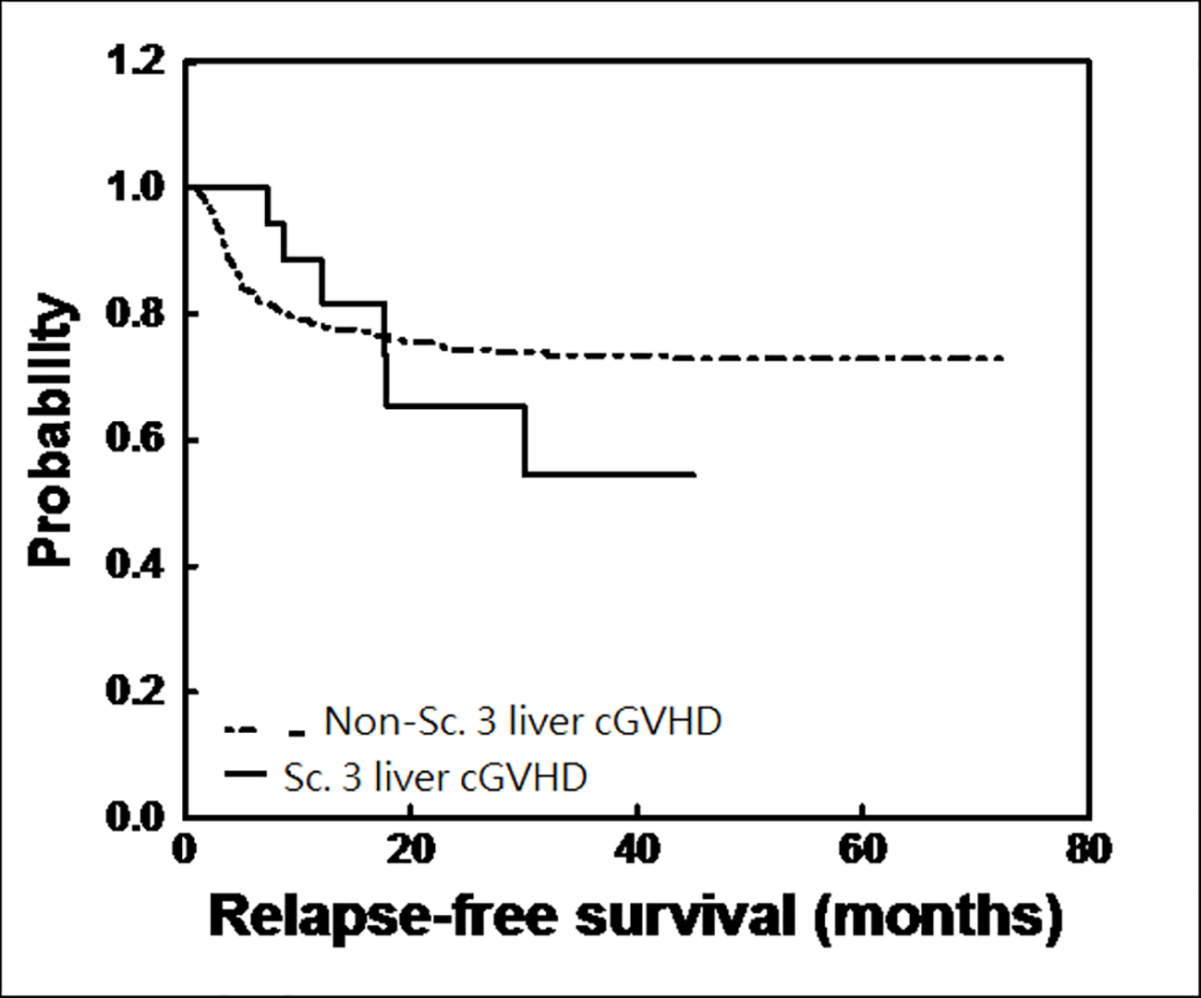
Relapse-free survival (RFS) after HSCT for patients with score 3 liver cGvHD or not. The relapse rate was comparable (23% vs 25%), giving it a non-significant difference of RFS (median RFS, 14.9 versus 13.8 months, HR 0.955, P = 0.914).

**Fig 3:**
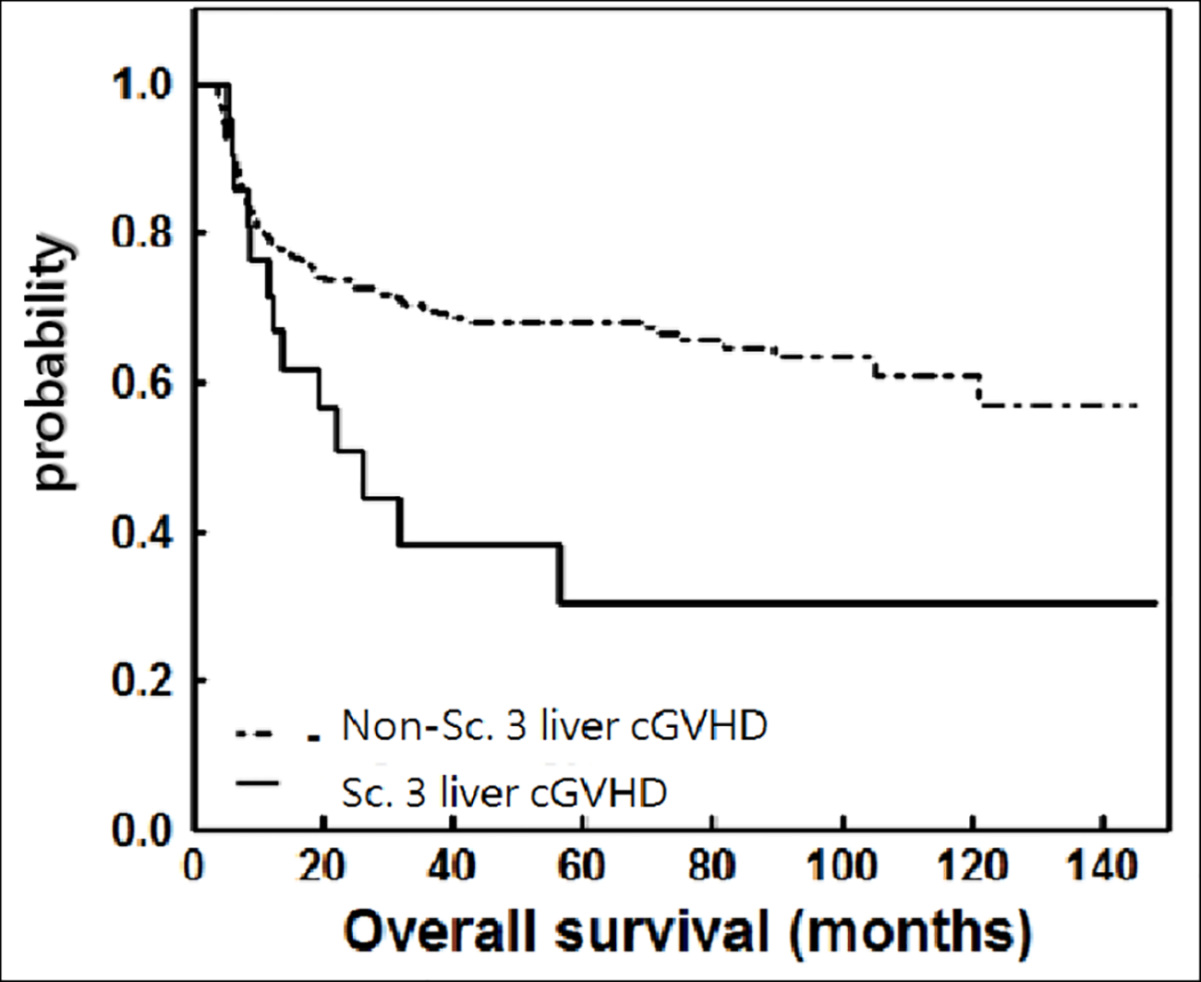
Overall survival (OS) after HSCT for patients developing score 3 liver cGvHD or not. There was a statistically significant difference in OS (median OS, 37 vs 19.4 months, HR 2.037, P = 0.019).

The impact of liver cGvHD scores of 3 on survival was adjusted in multivariable Cox regression analysis (Table in S2 Table). In the score 3 group, there were 6 relapses and 13 mortalities, including 4 disease-related and 9 non-relapse deaths. All relapses (6/6) occurred before 31^th^ month, compared to 23^th^ month for most relapses (84/87) in the non-3 group. The relapse rate was comparable (23% vs 25%), giving it a non-significant difference of relapse-free survival (median RFS, 14.9 versus 13.8 months, HR 0.955, 95% CI 0.411–2.217, P = 0.914) (Fig 2). However, there was a statistically significant difference in survival (median OS, 37 vs 19.4 months, HR 2.037, 95% CI 1.123–3.696, P = 0.019) between patients with score 3 liver cGvHD and those without (Fig 3). The OS curve had a steady decline until 43^th^ months. Most mortalities (102/109) occurred by this point.

## Discussion

In our analysis, the incidence of liver cGvHD scores of 3 was 5.8%, slightly lower than that reported by Pidala et al[16] (8%) and Bresters et al[24] (8%) in pediatric patients. Using 2005 NIH criteria, Pidala et al[16] reported liver cGvHD to be a poor prognostic factor for OS (HR 2.46, 95% CI 1.48–4.09, P = 0.001) and higher NRM (HR 2.15, 95% CI 1.13–4.11, P = 0.02). Comparing patients with score 3 cGvHD to those with score of less than 3 cGvHD, there was no difference in RFS but significant OS difference (Figs 2 & 3), after considering competing risk factors, including whether patients received second transplant (Table in S2 Table). In the group with score of less than 3, patients might tolerate better to salvage chemotherapy or lymphocyte infusion, which might contribute to more durable post-relapse survival and to significant OS difference.

The potential risk factors of post-transplant liver dysfunction in pediatric patients months or years after undergoing allo-HSCT include pre-transplant liver injury[25] and underlying benign hematological disease[24]. The former study did not analyze HCV carrier as a risk factor, while another showed non-significant findings due to a limited number of patients with HCV (N = 3). However, in an analysis of a Japanese transplant registry database, HCV positivity was associated with higher NRM, inferior OS in patients undergoing allo-HSCT[26, 27], and was a risk factor for acute liver GvHD in adults[28]. Thus, HCV carrier status has been persistently implicated in both liver aGvHD and cGvHD. Indeed, HCV is associated with immune dysfunction[29] and has been shown to prime the liver with a pro-inflammatory environment[27]. Though there is no consensus if HCV carriers should be treated with novel antiviral agents (Ledipasvir/Sofosbuvir) prior to transplant, we suggest that viral eradication might mitigate the risks of score 3 disease. Furthermore, for patients with pre-existing HCV infection and post-transplant hepatic dysfunction, liver biopsy help in differentiate abrupt onset of liver cGvHD from acute viral hepatitis[17].

The F-M donor-recipient gender combination is a risk factor for grade II to grade IV acute GvHD[30, 31] (aGvHD) and cGvHD[30, 32]. In our analysis, the predictive effect of F-M donor-recipient combination for score 3 liver cGvHD was offset after adjusting for male gender in multivariable analysis. The small sample size might prevent several established risk factors (F-M combination, MAC preparations, etc) from reaching statistical significance. Host innate immunity in different gender might play a role. A xenograft model showed clear sex differences in intestinal and peripheral innate immune cell populations[33].

Inconsistent with previous studies[34, 35], our results indicate that recipients less than 30 years of age tended to have a higher incidence of liver cGvHD. Lim et al.[36] hypothesized that the age-related variation in thymoglobulin pharmacokinetics may play a role in these findings, though the result was inconclusive. In our practice, physicians tend to maintain some degree of cGvHD without increasing cyclosporine dosage, especially in younger patients with higher-risk disease, which could partly explain our findings.

Apart from Pidala et al[16], who reported poorer OS (HR 3.73, P < 0.01) in patients with jaundice-type liver cGvHD (scores of 2 and 3 by 2014 NIH criteria), our results suggest lower OS in the group with score of 3, which might be driven by more durable post-relapse survival in the non-3 group. In this category, patients probably had higher potential of immune suppression and organ injury, making them prone to treatment related death after relapse. However, we have several limitations in our study, including the limited number of patients receiving bone marrow transplants, few HCV carriers, scarce relapse events, and the possible underestimation of GvHD prevalence in the setting of out-patient visits. The associations between liver dysfunctions and drugs or parenchymal liver disease were difficult to prove in the absence of adequate biopsy, and might introduce bias in identifying liver GvHD. The histologic changes of HCV infection share many features with those of liver GvHD, especially when fibrosing cholestatic hepatitis is a possibility [37, 38]. During data collection, there may have been an overlap between acute and chronic liver GvHD, despite differentiation based on the 2014 NIH criteria. In addition, we lacked complete data on cyclosporine concentrations throughout the GvHD course, HLA alleles C/DQ, and causes of death.

In our study, patients in the earlier era were not analyzed because of incoherent follow-up on liver function panel, ambiguous depiction of symptoms and signs relating to cGvHD in each patient’s visit, and missing record on transplant-related information. Thus, our result should be interpreted cautiously.

## Conclusion

Based on 2014 NIH consensus criteria, patients with liver cGvHD with a score of 3 had inferior outcome in overall survival. HCV carrier status and male gender were risk factors of developing cGvHD. Early recognition of this devastating complication might help in prompt immunosuppressive therapy and reducing late poor outcome.

## Supporting information

S1 Table Characteristics of 6 HCV carriers. (DOCX)

S2 Table Impact of score 3 liver cGvHD on overall survival. (DOCX)
