# Correction: Characteristics and risk of chronic graft-versus-host disease of liver in allogeneic hematopoietic stem cell transplant recipients

**DOI:** 10.1371/journal.pone.0197797

**Published:** 2018-05-17

**Authors:** Chien-Ting Chen, Chun-Yu Liu, Yuan-Bin Yu, Chia-Jen Liu, Liang-Tsai Hsiao, Jyh-Pyng Gau, Tzeon-Jye Chiou, Jing-Hwang Liu, Yao-Chung Liu

The first two authors, Chien-Ting Chen and Chun-Yu Liu, should not have been attributed equal contribution to this work.
