# Supplementary material for: Characteristics and risk of chronic graft-versus-host disease of liver in allogeneic hematopoietic stem cell transplant recipients
Source: PLoS One. 2017 Sep 21;12(9):e0185210. doi: 10.1371/journal.pone.0185210 (PMC5608321; doi:10.1371/journal.pone.0185210)
Supplement: S1 Table — (DOCX) [file pone.0185210.s001.docx]

|  | | | | |
| --- | --- | --- | --- | --- |
| patient | HCV titer (10^6^ IU/ml) | | Pathology of liver biopsy | liver cGvHD |
|  | Pre-HSCT | Post-HSCT |  |  |
| 1 | low titer | low titer | NP | no |
| 2 | 53.4 | 15.3 | NP | no |
| 3 | 4.91 | 3.97 | Mild portal area inflammatory cells infiltration, bile duct degeneration | yes, score 3 |
| 4 | NA | NA | NP | no |
| 5 | NA | NA | NP | no |
| 6 | NA | NA | Bile duct degeneration, mild endothelialitis, T cells infiltration in portal area, diffuse sinusoidal fibrosis | yes, score 3 |
| HCV, hepatitis C virus; HSCT, hematopoietic stem cell transplant; cGVHD, chronic graft-versus-host disease; NA, not analysed; NP, not performed | | | | |
