# Supplementary material for: Characteristics and risk of chronic graft-versus-host disease of liver in allogeneic hematopoietic stem cell transplant recipients
Source: PLoS One. 2017 Sep 21;12(9):e0185210. doi: 10.1371/journal.pone.0185210 (PMC5608321; doi:10.1371/journal.pone.0185210)
Supplement: S2 Table — (DOCX) [file pone.0185210.s002.docx]

|  | | | | | | | |
| --- | --- | --- | --- | --- | --- | --- | --- |
|  |  | **Mortality** | | **Univariate** |  | **Multivariate** |  |
| **Risk factor** | **Patient (*N*)** | ***N*** | **%** | **HR (95% CI)** | **P** | **HR (95% CI)** | **P** |
| **Gender** |  |  |  |  |  |  |  |
| female | 149 | 43 | 39.4 | 1.00 (reference) |  |  |  |
| Male | 213 | 79 | 37.1 | 1.350 (0.931–1.957) | 0.114 |  |  |
| **Age** |  |  |  |  |  |  |  |
| ＜ 30 | 125 | 32 | 25.6 | 1.00 (reference) |  |  |  |
| ≧ 30 | 237 | 90 | 39.0 | 1.704 (1.137–2.533) | 0.010 | 1.443 (0.947–2.198) | 0.088 |
| **Diagnosis** |  |  |  |  |  |  |  |
| Non-malignant | 54 | 6 | 11.1 | 1.00 (reference) |  |  |  |
| Malignant | 308 | 116 | 37.7 | 3.848 (1.693–8.746) | 0.001 | 2.787 (1.210–6.415) | 0.016 |
| **Transplant type** |  |  |  |  |  |  |  |
| Non-MSD | 185 | 62 | 33.5 | 1.00 (reference) |  |  |  |
| MSD | 176 | 60 | 34.1 | 1.035 (0.725–1.477) | 0.852 |  |  |
| **EBMT score** |  |  |  |  |  |  |  |
| ≦2 | 170 | 38 | 22.4 | 1.00 (reference) |  |  |  |
| ＞2 | 192 | 84 | 43.8 | 2.437 (1.657–3.584) | 0.000 | 1.945 (1.296–2.918) | 0.001 |
| **Intensity of conditioning regimen** |  |  |  |  |  |  |  |
| Reduced-intensity | 118 | 30 | 25.4 | 1.00 (reference) |  |  |  |
| Myeloablative | 244 | 92 | 37.7 | 1.539 (1.019–2.324) | 0.041 | 1.419 (0.909–2.217) | 0.124 |
| **Transplant number** |  |  |  |  |  |  |  |
| 1 | 333 | 109 | 32.7 | 1.00 (reference) |  |  |  |
| ＞1 | 29 | 13 | 44.8 | 1.798 (1.029–3.141) | 0.039 | 1.144 (0.630–2.076) | 0.658 |
| **Score 3 liver cGvHD** |  |  |  |  |  |  |  |
| No | 341 | 109 | 42.0 | 1.00 (reference) |  |  |  |
| Yes | 21 | 13 | 61.9 | 2.056 (1.155–3.663) | 0.014 | 2.037 (1.123–3.696) | 0.019 |
|  | | | | | | | |
